# Inotuzumab ozogamicin for relapse prevention in a boy with Down syndrome and relapsed acute lymphoblastic leukemia

**DOI:** 10.1007/s12185-024-03890-1

**Published:** 2024-12-09

**Authors:** Atsushi Kohso, Hidemi Toyoda, Ryo Hanaki, Kaori Niwa, Yosuke Okumura, Mari Morimoto, Takahiro Ito, Masahiro Hirayama

**Affiliations:** Department of Pediatrics, Mie University Graduate School of Medicine, 2-174 Edobashi, Tsu, Mie 514-8507 Japan

**Keywords:** B-cell precursor acute lymphoblastic leukemia, Relapse, Inotuzumab ozogamicin, Maintenance chemotherapy

## Abstract

Inotuzumab ozogamicin (InO), a CD22-directed antibody conjugated to calicheamicin, has demonstrated excellent efficacy in B-cell precursor (BCP) acute lymphoblastic leukemia (ALL). It has been used for patients with relapsed or refractory BCP-ALL as a bridge to allo-HCT. Children with Down syndrome (DS) have an increased risk of BCP-ALL and higher rates of relapse and toxicity, including treatment-related mortality. Although allo-HCT is potentially curative for relapsed or refractory ALL, post-transplant leukemic relapse rates and transplant-related mortality are dismal in patients with DS-ALL, which results in less frequent use of allo-HCT in this group than in the non-DS population. Therefore, novel and less toxic therapeutic strategies are required to improve outcomes. Here we report the case of a child with DS who was diagnosed with a second relapse of BCP-ALL and has maintained complete remission through regular single-agent InO therapy. Single-agent maintenance using InO can be a good option to avoid subsequent relapse in patients with relapsed or refractory BCP-ALL who cannot proceed to allo-HCT and require less-toxic treatments.

## New findings

Inotuzumab ozogamicin (InO), an anti-CD22 monoclonal antibody bound to calicheamicin, has significant activity against BCP-ALL and has been used for patients with relapsed or refractory BCP-ALL as a bridge to allogeneic hematopoietic transplantation (allo-HCT). However, allo-HCT is not suitable for patients at high risk of treatment-related toxicity, including those with Down syndrome (DS). We describe a boy with second relapsed DS-associated BCP-ALL maintaining complete remission with regular InO administration. InO-mediated maintenance therapy may be a good option to avoid subsequent relapse in patients with relapsed or refractory BCP-ALL who cannot proceed to allo-HCT and require less-toxic therapeutic strategies.

## Introduction

Children with Down syndrome (DS) have an increased risk of B-cell precursor (BCP) acute lymphoblastic leukemia (ALL). Patients with DS-ALL face a dismal prognosis, because of higher treatment-related mortality (TRM) and increased rates of relapse compared to patients with non-DS-ALL [1–3]. Although most relapsed DS-ALL cases are late relapses, which have more favorable characteristics than early relapses, the post-relapse overall survival of relapsed DS-ALL remains dismal compared with non-DS-ALL (18.3% ± 11.7% vs. 63.9% ± 2.7%) [4]. Allogeneic hematopoietic stem cell transplantation (allo-HCT) is a potentially curative approach for managing relapsed or refractory ALL. However, given the high rates of post-transplant leukemic relapse and transplant-related mortality (54% and 22%, respectively), allo-HCT is used less often in the DS-ALL patients than in the non-DS population [2]. Given that patients with relapsed DS-ALL often cannot tolerate intensive salvage strategies, post-remission therapies to mitigate subsequent relapse without allo-HCT are required to improve outcomes.

Inotuzumab ozogamicin (InO) is a CD22-targeted monoclonal antibody bound to calicheamicin, which has been shown to have significant activity against BCP-ALL [5]. The randomized phase 3 INO-VATE trial showed an overall complete remission (CR) rate of 81% in the InO arm as compared with 29% in the standard arm [5]. Based on these data, InO has been approved for the treatment of relapsed or refractory CD22-positive BCP-ALL in adults in the USA, Europe, Japan and several other countries worldwide. In March 2024, InO received its first pediatric approval in the USA and Japan for this indication in patients aged ≥ 1 year. InO has been administered to patients with relapsed or refractory BCP-ALL as a bridge to allo-HCT. Although children treated with InO had high response rates of 58.3–81.5%, sinusoidal obstructive syndrome (SOS) occurred in 25.0–28.6% of these patients, particularly when InO treatment was followed by allo-HCT [6, 7]. Recently, evidence has emerged suggesting the effectiveness of lower doses of InO to prevent post-transplant relapse of BCP-ALL in adults [8].

We hypothesized that low-dose InO would be safe and feasible for relapse prevention in patients with relapsed or refractory BCP-ALL who were heavily pretreated and intolerant of allo-HCT. Herein, we report a patient experiencing a second relapse of DS-ALL who achieved a third CR by bortezomib-combined induction therapy and maintained CR by regular administration of single-agent InO without allo-HCT.

## Case report

A 3-year-old male with DS and a surgical history of a ventricular septal defect repair at 2 months of age was admitted with fever and leg pain. Laboratory results showed a white blood cell (WBC) count of 6.1 × 10^9^/L with 17% blast cells. Bone marrow (BM) evaluation showed 74% blast cells with lymphoid characteristics. Immunophenotyping confirmed the diagnosis of BCP-ALL. Fluorescence in situ hybridization (FISH) revealed *ETV6::RUNX1* rearrangement. No extramedullary organ infiltration was observed. The patient was treated according to the standard risk group protocol of Japan Association of Childhood Leukemia Study ALL-02 [9]. Given the increased risk of high-dose methotrexate-associated toxicity in children with DS-ALL, a reduced dose of methotrexate (1.5 g/m^2^ over 24 h instead of 3.0 g/m^2^) was given intravenously. The number of leukemic blasts in the peripheral blood was reduced after one week of prednisolone monotherapy, and BM examination after induction therapy revealed CR. Although the patient had been clinically well, he presented with a persistent fever at 40 months after primary diagnosis (15 months after completion of frontline therapy). At this time, the patient had a WBC count of 4.2 × 10^9^/L with 0% blast cells, while morphologic BM evaluation revealed 65% lymphoid blast cells with *ETV6::RUNX1* signals. No extramedullary organ infiltration was observed. He was diagnosed with late isolated BM relapse of BCP-ALL and was treated according to the ALL-REZ BFM-2002 Protocol II-IDA regimen [10]. The initial high-dose methotrexate was reduced from 1.0 g/m^2^ over 36 h to 0.5 g/m^2^ over 36 h, however since no methotrexate-associated toxicity was observed, the usual dose (1.0 g/m^2^ over 36 h) was administered after the second high-dose methotrexate therapy. He achieved a second CR after induction chemotherapy, and polymerase chain reaction (PCR) of the BM yielded negative results for measurable/minimal residual disease (MRD). Therefore, the patient had been treated by chemotherapy alone, without allo-HCT.

**Fig. 1:**
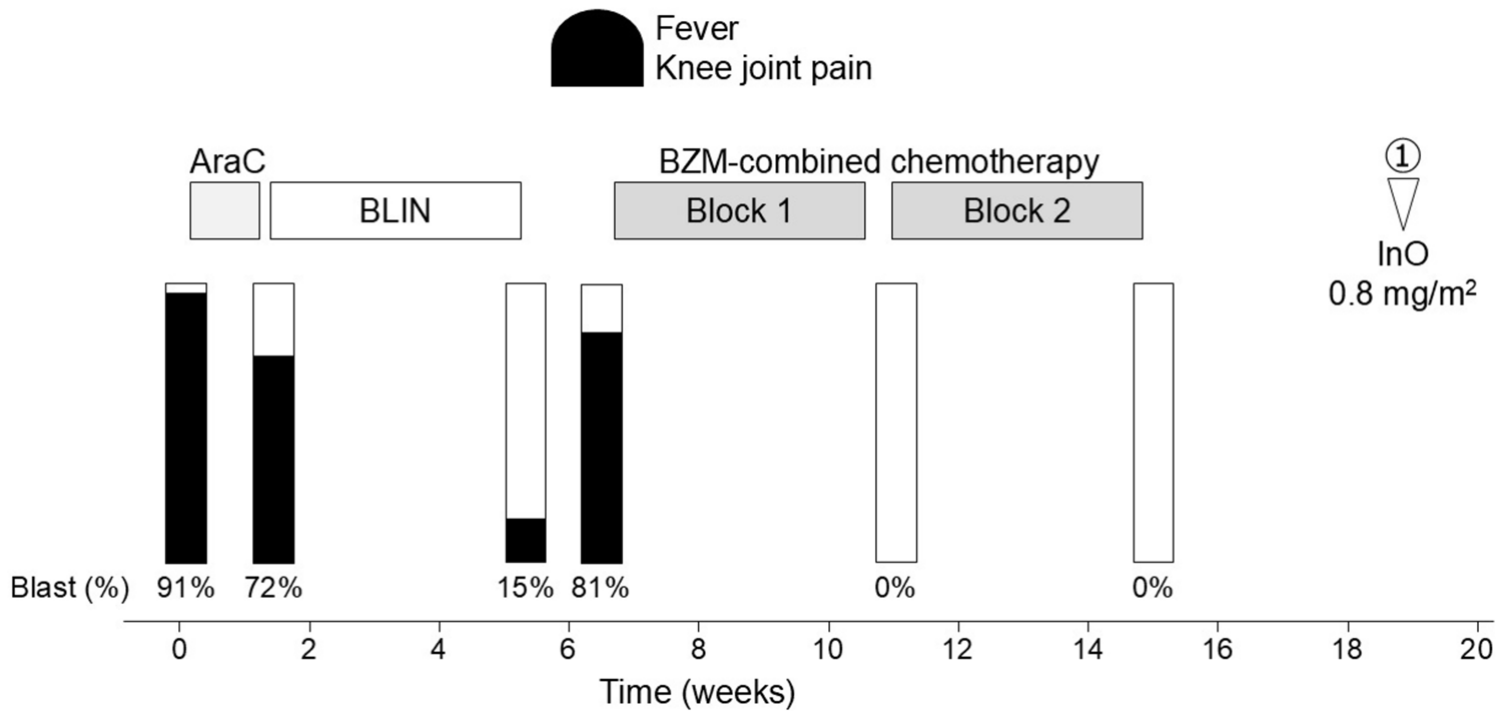
Clinical course of the patient with second relapsed DS-ALL. BLIN, blinatumomab; BZM, bortezomib; InO, inotuzumab ozogamycin

**Fig. 2:**
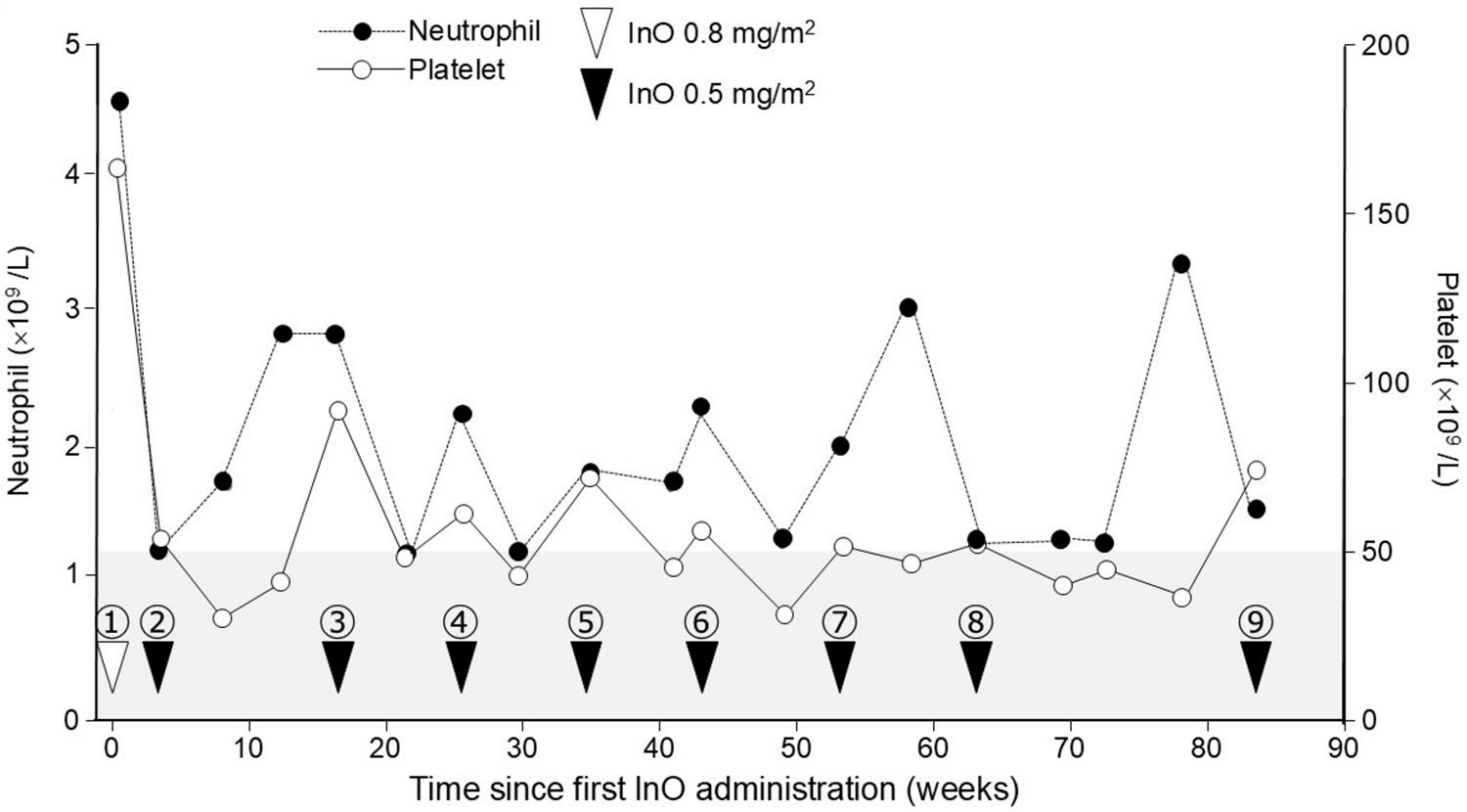
Clinical course of the patient from the inotuzumab ozogamicin initiation. Areas with platelet levels below 50 × 10^9^ /L are highlighted by gray shading. Open triangle, inotuzumab ozogamycin 0.8 mg/m^2^; filled triangle, inotuzumab ozogamycin 0.5 mg/m^2^; InO, inotuzumab ozogamycin

Although he had been clinically well since achieving second CR, at the age of 12, he presented with persistent fever and leg pain at 77 months after diagnosis of first relapse (37 months after completion of relapse therapy). Morphologic evaluation and FISH analysis of the BM revealed 91% lymphoid blast cells with *ETV6::RUNX1* rearrangement. Because no extramedullary organ infiltration was observed, he was diagnosed with a second isolated BM relapse of BCP-ALL. After partial cytoreduction with cytarabine (20 mg/m^2^ daily for 5 days), single-agent blinatumomab was administered for 4 weeks; however, 81% of the lymphoid blasts positive for CD19 were still observed in the BM (Fig. 1). Therefore, bortezomib-combined chemotherapy was initiated as previously reported (Fig. 1) [11]. Bortezomib, 1.3 mg/m^2^, was administered as 7 intravenous doses on days 1, 4, 8 and 11 in Block 1 and on days 1, 4, and 8 in Block 2. Block 1 consisted of a standard VPLD 4-drug induction with bortezomib. Prednisone (40 mg/m^2^/d) was given intravenously for 28 consecutive days. Doxorubicin (60 mg/m^2^) was given intravenously on day 1. Vincristine (1.5 mg/m^2^) was administered intravenously on days 1, 8, 15 and 22. L-asparaginase (10,000 U/m^2^) was given intravenously twice a week for 8 doses instead of pegylated asparaginase. Intrathecal methotrexate was administered on days 1, 15 and 29. Block 2 was a combination of cyclophosphamide and etoposide with bortezomib followed by high-dose methotrexate. Five doses of both etoposide (100 mg/m^2^) and cyclophosphamide (440 mg/m^2^) were given intravenously from days 1 to 5. Given the increased risk of high-dose methotrexate-associated toxicity in children with DS-ALL, a reduced dose of methotrexate (500 mg/m^2^) was given intravenously on day 22 of Block 2. Intrathecal methotrexate was administered on days 1 and 22. After Block 2, the patient achieved a third CR with a negative PCR-MRD. The patient experienced grade 4 hematological toxicity, but non-hematological toxicities, including infections, never reached grade 3. Although curative allo-HCT or chimeric antigen receptor (CAR)-T cell therapy was offered, the patient and his parents refused because of the poor prognosis and TRM of relapsed DS-ALL. Therefore, treatment with single-agent InO, 0.8 mg/m^2^ was initiated as a subsequent chemotherapy on day 35 after Block 2 of bortezomib-combined regimen. InO is usually administered as follows: 0.8 mg/m^2^ on day 1, followed by 0.5 mg/m^2^ on days 8 and 15 of an initial course, with the courses being repeated every 3 to 4 weeks [5]. However, a second dose was administered 4 weeks after the initial InO treatment due to prolonged thrombocytopenia (Fig. 2). Although subsequent InO treatments also needed to be delayed (Fig. 2), the patient has been in CR for the last 20 months following InO initiation, with no toxicity besides prolonged thrombocytopenia. However, the patient had his first relapse 15 months after completing frontline therapy and his second relapse 37 months after completing relapse therapy. Therefore, a 20-months follow-up period is not a sufficient follow-up period and close observation for subsequent relapse is required.

## Discussion

In this study, we described a patient with a second relapse of DS-ALL who maintained CR by regular administration of InO as a single-agent treatment. Patients with DS-ALL have a high incidence of TRM and relapse [1, 4]. Although allo-HCT is a potentially curative approach to manage relapsed or refractory ALL, given the high probability of post-transplant leukemic relapse and transplant-related mortality, the frequency of use of allo-HCT remains less in DS-ALL [2]. Although maintenance therapy is essential for curing ALL, post-remission maintenance therapies to mitigate subsequent relapse without allo-HCT have not been established for relapsed DS-ALL.

Prophylactic administration of InO has been investigated as a strategy to reduce ALL relapse after allo-HCT in adults with high risk of relapse [8]. Low-dose InO, when administered as a relapse prevention therapy after allo-HCT, offers a high one-year progression-free survival rate (89%), has a favorable safety profile, and is not associated with excess toxicity in clinical applications, with no observed SOS or graft failure [8]. Furthermore, lower doses of InO were used in adult patients with Philadelphia chromosome-positive or -negative ALL who did not achieve MRD negativity after frontline or salvage therapy resulting in favorable survival, MRD negativity rates, and safety profiles for patients with ALL and MRD-positive status [12]. Notably, Brivio et al. reported that one patient showed prolonged continuous CR for 15 months after two courses of InO without any additional treatment [13]. They also reported that two other patients were in continuous CR for 19 months after InO treatment followed by chemotherapy or blinatumomab, although they did not undergo allo-HCT [13]. Based on the impressive outcomes seen in relapsed or refractory setting and favorable toxicity profile of single-agent InO compared to intensive chemotherapy, we hypothesized that InO would be a feasible agent for subsequent relapse prevention in patients with BCP-ALL who were heavily pretreated and intolerant to allo-HCT. InO is administered at 1.8 mg/m^2^ per cycle, 0.8 mg/m^2^ on day 1 and 0.5 mg/m^2^ on days 8 and 15 of a 21- to 28-day cycle until CR is achieved [5]. After CR, subsequent cycles are given at 1.5 mg/m^2^ per cycle, 0.5 mg/m^2^ on days 1, 8 and 15 of a 28-day cycle; the patients receive treatment for up to six cycles [5]. Absolute neutrophil count > 1.0 × 10^9^/L and a platelet count > 50 × 10^9^/L for 7 days are required for InO administration. We intended to perform InO treatment according to schedule; however, because of its hematological toxicity, especially thrombocytopenia, we had to lengthen the interval between InO doses from 4 to 10 weeks (Fig. 2), resulting in a treatment approach similar to that for post-transplant relapse prevention trial using InO [8]. Although thrombocytopenia requires lengthening the InO dose interval, we plan to administer a maximum of 6 cycles of 3 doses per cycle, since long-term maintenance therapy is effective for preventing subsequent relapse. In contrast, since Metheny et al. reported that even low-doses InO can eliminate MRD [8], it is worth considering reducing the InO dose to 0.3 mg/m^2^/dose, given the prolonged thrombocytopenia in the patient induced by InO. Since the patient achieved PCR-MRD negative CR with two cycles of bortezomib-combined chemotherapy before the InO treatment, it is unclear whether the patient’s relapse-free status was due to the effect of InO treatment or of bortezomib-combined chemotherapy. However, considering that the patient would have relapsed early if post-induction therapy had not been administered, InO-mediated maintenance therapy was considered to help preventing subsequent relapse. In our case, CAR-T cell therapy or allo-HCT were not chosen because of the high therapeutic toxicity in ALL with DS. Therefore, it should be noted that InO-mediated maintenance therapy was tolerated well in this patient with second relapsed DS-ALL, and no adverse events beside prolonged thrombocytopenia were observed.

This study does not answer one critical question: what cycles, intervals, and doses of InO can safely and feasibly be given for relapse prevention? Although a maximum of six cycles are allowed, it is not clear a priori how many cycles and doses of InO would be safe and effective in reducing relapse in patients with relapsed or refractory BCP-ALL who cannot proceed to allo-HCT. To answer this question, a prospective large-scale study is needed to further investigate the most feasible and safe schedule for InO administration.

Our study demonstrated that regular InO administration may prevent and overcome subsequent relapse without allo-HCT in patients with relapsed or refractory DS-ALL, who require less toxic therapeutic strategies than other patients with ALL, to improve outcomes. Regular administration of InO with longer dosing intervals may be a good option to avoid subsequent relapse in patients with relapsed or refractory BCP-ALL who cannot proceed to intensive chemotherapy or allo-HCT.

## Data Availability

The data that support the findings of this study are not openly available due to reasons of sensitivity and are available from the corresponding author upon reasonable request.
